# HLA variations in patients with diffuse large B-cell lymphoma and association with disease risk and prognosis: a case-control study

**DOI:** 10.3389/fgene.2024.1341822

**Published:** 2024-04-12

**Authors:** Ioanna Diamanti, Asimina Fylaktou, Evgenia Verrou, Efthimia Vlachaki, Manolis Sinakos, Eirini Katodritou, Konstantinos Ouranos, Fani Minti, Georgia Gioula

**Affiliations:** ^1^ Biochemisty and Microbiology Department, Theagenio Cancer Hospital, Thessaloniki, Greece; ^2^ National Peripheral Histocompatibility Center-Immunology Department, Hippokration General Hospital, Thessaloniki, Greece; ^3^ Hematology Department, Theagenio Cancer Hospital, Thessaloniki, Greece; ^4^ Thalassemia Unit-2nd Department of Internal Medicine, Hippokration General Hospital, School of Medicine, Faculty of Health Sciences, Aristotle University of Thessaloniki, Thessaloniki, Greece; ^5^ 4th Department of Internal Medicine, Hippokration General Hospital, School of Medicine, Faculty of Health Sciences, Aristotle University of Thessaloniki, Thessaloniki, Greece; ^6^ Department of Medicine, Houston Methodist Research Institute, Houston, TX, United States; ^7^ Microbiology Department, School of Medicine, Faculty of Health Sciences, Aristotle University of Thessaloniki, Thessaloniki, Greece

**Keywords:** HLA alleles, genetic polymorphisms, DLBCL, risk, lymphoma

## Abstract

**Introduction:**

Human leukocyte antigen (HLA) polymorphisms have been associated with the development of various autoimmune diseases, as well as malignant neoplasms. Non-Hodgkin lymphomas (NHLs) are a heterogenous group of lymphoid malignancies in which a genetic substrate has been established and is deemed to play a crucial role in disease pathogenesis. This study aimed to identify whether variations in the HLA gene region were associated with diffuse large B-cell lymphoma (DLBCL) risk and prognosis.

**Methods:**

We defined HLA class I (HLA-A, HLA-B, HLA-C) and class II (HLA-DRB1, HLA-DQB1) alleles in 60 patients with DLBCL and compared the results to those found by 236 healthy adult donors from the bone marrow bank of Northern Greece. HLA typing was performed by two molecular methods, Sequence - Specific Oligonucleotide HLA typing (SSO) and Sequence - Specific Primer HLA typing (SSP), from white blood cells recovered from peripheral blood. The phenotypic frequencies of HLA-A, HLA-B, HLA-C, HLA-DRB1 and HLA-DQB1 between patients and controls were compared with the 2-sided Fisher’s exact test. Results with *p*-value <0.05 were considered statistically significant. Odds Ratios with 95% Confidence Intervals were calculated to further strengthen the results. The 2-sided Fisher’s exact test was also applied to alleles found only in one of the two groups, while the odds ratios together with the confidence intervals were corrected with Haldane-Anscombe method.

**Results:**

Among the studied HLA polymorphisms, the frequency HLA-C*12 allele was significantly lower in patients with DLBCL compared with control subjects (6.7% vs. 34.7%, OR = 0.16, 95% CI: 0.04–0.44). Frequency of HLA-B*39 was significantly lower in patients with DLBCL compared with controls, but due to the low frequency of this polymorphism in the studied population and small sample size, determinations regarding the significance of this findings were limited. Survival analysis revealed that the presence of HLA-C*12 was not associated with improved or worsened overall and progression-free survival. No statistically significant associations were observed in the phenotypic frequencies of HLA-A, HLA-DQB1, HLA-DRB1 and the rest of HLA-B alleles between the control and DLBCL groups.

**Discussion:**

Collectively, our results provide valuable insight regarding the role of HLA variations on DLBCL risk. Further studies are required to consolidate our findings and ascertain the clinical implications of these genetic variations on DLBCL management and prognosis.

## 1 Introduction

Diffuse large B-cell lymphoma (DLBCL) is the most common subtype of non-Hodgkin lymphoma (NHL), comprising 30% of the total NHL cases in Western countries (Thandra et al., 2021). Although the etiology of this hematologic malignancy is multifactorial, recent genome-wide association studies (GWAS) have revealed specific genetic loci serving as potential substrates that drive the development of DLBCL (Cerhan et al., 2014; Chihara et al., 2015; Hernández-Verdin et al., 2020). This genetic component is further reinforced by the observation that the incidence rate of DLBCL varies considerably among races and ethnicity. These regional differences may be partly explained by distinct polymorphisms in genes responsible for immune surveillance and tumoral spread control, as evidenced by studies including populations of different ethnicities that analyze genetic susceptibility to DLBCL (Choi et al., 2008; Bassig et al., 2015).

Gene variations associated with the pathogenesis of DLBCL have been described in the literature, with the Human Leukocyte Antigen (HLA) genes encoded in the Major Histocompatibility Complex at chromosome 6p21.3, being of primary importance. Structural or functional alterations of the HLA molecule may prevent the tumoral antigen from being presented to cytotoxic T cells, thereby evading the host’s immune response. As a result, unchecked malignant cell proliferation and tumoral spread ensue. These alterations may be due to mutations in genes encoding the alpha chain in HLA class I or alpha and beta chains in HLA class II molecules (Fangazio et al., 2021), or due to inactivating mutations in genes encoding for proteins necessary for proper HLA structure and function, such as beta-2 microglobulin of HLA class I and the invariant chain of HLA class II molecules, leading to dysregulation and/or downregulation of the HLA molecule (Pasqualucci and Dalla-Favera, 2018; Fangazio et al., 2021). HLA-I downregulation, specifically, has been associated with higher neoantigen load in various cancer types (McGranahan et al., 2017), including lymphomas (Fangazio et al., 2021). In DLBCL, HLA-I loss is more commonly observed than HLA-II loss (McGranahan et al., 2017; Nijland et al., 2017; Pasqualucci and Dalla-Favera, 2018). Also, mutations leading to the aberrant location, mostly cytoplasmic, of the HLA molecule, prevent its transfer to the cell surface, impeding its role in antigen presentation (Pasqualucci and Dalla-Favera, 2018). Another HLA-associated mechanism that has been associated with DLBCL etiology, is HLA diversity. Homozygosity for various HLA loci reduces the available antigenic repertoire for presentation to T-cells, some of which may be tumor-derived, and thus, prevents proper immune surveillance from occurring, and facilitates tumor spread (Fangazio et al., 2021). Except for impacting the risk of DLBCL development, HLA polymorphisms can affect an individual’s response to treatment and confer worse long-term prognosis (Lu et al., 2011; Alcoceba et al., 2013; Higashi et al., 2016).

The advent of GWAS has brought to attention specific HLA variations that were not detected by previous methodological techniques that were harnessed to study HLA molecules and DLBCL risk and prognosis (Smedby et al., 2011; Ghesquieres et al., 2015; Zhong et al., 2019; Hernández-Verdin et al., 2020). Most of these polymorphisms increase, although some have been found to decrease, the risk of developing a specific NHL subtype (Zhong et al., 2019). Distinct HLA alleles are usually related to specific NHL subtypes, although pleiotropic HLA associations have been described (Smedby et al., 2011). Some of these associations were observed in studies that included populations from a single race/ethnicity, although subsequent studies examining the association of similar HLA variations in a cohort of a different race/ethnicity, corroborated the results (Alcoceba et al., 2013; Bassig et al., 2015; Ten et al., 2017; Zhong et al., 2019). Nevertheless, the impact of regional HLA variations on the risk of DLBCL development remains to be elucidated in future genomic studies. A meaningful insight into the frequency of specific HLA variations among patients with DLBCL would enhance our understanding of disease pathogenesis and possibly yield more information about the quantification of risk and prognosis of this aggressive malignancy.

In the present study, we evaluated the prevalence of specific HLA polymorphisms in a series of 60 DLBCL patients and we compared the results to those of healthy individuals in order to assess whether HLA variations are associated with DLBCL risk. We then aimed to ascertain whether HLA polymorphisms found to be significantly associated with DLBCL risk could affect the prognosis in patients diagnosed with this malignancy.

## 2 Materials and methods

### 2.1 Study design

We conducted a single-center case-control study to ascertain the impact of HLA polymorphisms on DLBCL risk and prognosis. We defined HLA class I (HLA-A, HLA-B, HLA-C) and class II (HLA-DRB1, HLA-DQB1) alleles in patients with a diagnosis of DLBCL and a control group of healthy adult donors from the bone marrow bank of Northern Greece. Control group was age-, race-, and ethnicity-matched to the patients’ group. The study was approved by the Bioethics and Ethics Committee of Aristotle University of Thessaloniki (4.674/17.07.2019).

### 2.2 Study population

In this study, cases were defined as individuals with biopsy-proven diagnosis of DLBCL. Patients were genetically unrelated to each other. The diagnosis of DLBCL was made during the time period from 01/2002 to 12/2021, whereas data collection and specimen analysis occurred between 01/2018 and 12/2022. The control group consisted of healthy adult donors from the bone marrow bank of Northern Greece. Bone marrow donors were between 18 and 50 years old and they did not suffer from asthma, insulin-dependent diabetes, cancer, heart disease, multiple sclerosis, muscular dystrophy, schizophrenia, depression, infectious diseases, such as human immunodeficiency virus (HIV), hepatitis B and C, autoimmune disorders, vascular diseases, arterial or venous thrombosis and von Willebrand disease. In general, donors had a healthy medical record according to national guidelines for blood marrow donors. Individuals with a history of drug abuse, as well as those who were recipients of solid organ or hematopoietic cells in the past or were at risk for Creutzfeldt-Jacob disease, were excluded from the study. Healthy donors were recruited at the same time period when data specimen analysis for DLBCL patients occurred, in order to analyze specimens from both DLBCL patients and controls using the same technique, as described below. Upon study entry, the control group was considered healthy with no update request regarding their health status except if they were found to be compatible with a patient.

### 2.3 Study endpoints

The primary endpoint of the study was to assess whether patients with DLBCL have distinct HLA polymorphisms compared to the control group. As a secondary outcome, statistically significant associations were then examined to assess whether they confer worse or improved overall survival (OS) and/or progression-free survival (PFS) in patients with DLBCL.

### 2.4 HLA typing

The typing of HLA antigens was performed by two molecular methods, Sequence - Specific Oligonucleotide HLA typing (SSO) and Sequence - Specific Primer HLA typing (SSP). For the typing of HLA antigens, peripheral blood with EDTA anticoagulant was used. White blood cells were recovered from whole blood, and further genetic analysis was performed. Typing at low resolution level was performed by SSO (LIFECODE, Immucor GTI Diagnostics, Inc., United States of America), and, when required, typing was performed by SSP (PROTRANS, GERMANY).

SSO is a method based on the hybridization of specific oligonucleotide probes, which can be either DNA or RNA; DNA sequences are initially amplified by the polymerase chain reaction (PCR) method. First, a group of associated alleles is amplified with the appropriate primers, and then the amplified DNA is sequenced and hybridized with the panel of genetic probes that distinguishes the specific locus of the group of alleles that are under investigation. The Luminex technique uses polystyrene microbeads which are coated with different oligonucleotides. The tested DNA is amplified with PCR and subsequently hybridized. Then DNA is divided into a plate together with the beads that are coated with specific alleles. Hybridized DNA binds to the specific allele-coated microbeads, stained with phycoerythrin, a fluorochrome, and counted on the Luminex analyzer.

SSP uses pair of primers to identify conserved sequences. Both primers used in each PCR reaction match perfectly with the three edges of a single specific allele or group of alleles. During PCR, only those regions of the sample DNA that include the corresponding complementary sequences that perfectly match those of the primers are amplified. The amplified PCR product is detected by agarose gel electrophoresis. If the corresponding sequence is present in the sample, a band will be created in the gel. If the sample does not contain the corresponding sequence, there will be no amplification in the final product and there will be no bands in the agarose gel. Gel is analyzed by using the Primer and Amplification tables or Helmberg SCORE Software (Madden and Chabot-Richards, 2019; Jaramillo and Hacke, 2024).

### 2.5 Statistical analysis

Statistical analysis was conducted with the R programming language, version 4.2.2. The phenotypic frequencies of HLA-A, HLA-B, HLA-C, HLA-DRB1 and HLA-DQB1 between patients and controls were compared with the 2-sided Fisher’s exact test. Results with *p*-value <0.05 were considered statistically significant. Odds Ratios with 95% Confidence Intervals were calculated to further strengthen the results. The 2-sided Fisher’s exact test was also applied to alleles found only in one of the two groups, while the odds ratios together with the confidence intervals were corrected with Haldane-Anscombe method.

To investigate the association of the patients’ clinical-biological characteristics, including sex, age, stage, lactate dehydrogenase (LDH) levels, survival prognostic indices (revised International Prognostic Index [R-IPI], neutrophil-to-lymphocyte ratio [NLR] and lymphocyte-to-monocyte ratio [LMR], cell-of-origin status (activated B-cell like [ABC-like] DLBCL, germinal center B-cell-like [GCB-like] DLBCL, and not otherwise specified [NOS] DLBCL), relapse status and treatment regimen received with the OS and PFS, both univariate and multivariate survival analyses were conducted with the “survivalAnalysis” package, version 0.3.0 (Author Anonymous, 2024) of the R programming language. Specifically, Kaplan- Meyer plots were used for visualization of survival curves. Long-rank tests were used to detect differences in survival times between groups of patients based on patients’ clinical-biological characteristics and the presence or absence HLA polymorphism found to be significantly associated with DLBCL risk. Multivariate survival analysis was performed with the Cox proportional hazards model, to evaluate simultaneously the effect of patients’ clinical-biological characteristics that was characterized as statistically significant in univariate analysis, on survival. Results with *p*-values <0.05 characterized as statistically significant and 95% confidence intervals were calculated.

## 3 Results

### 3.1 Study population

HLA-A, -B, -C, DRB1, and DQB1 allele frequencies were analyzed and compared between 236 healthy control individuals and 60 patients with DLBCL. Briefly, control subjects were between the ages of 18 and 50 years (median age, 50 years). The number of healthy male subjects was 134 (56.8%), while 102 (43.2%) were female. Regarding patients with DLBCL, 33 (55%) were male and 27 (45%) were female. Median age of patients was 68.5 years. Analysis based on COO revealed that 21 (35.6%) patients had ABC-like DLBCL, 13 (22%) patients had GCB-like DLBCL, and 25 (42.4%) patients had DLBCL, NOS. All patients had *de novo* DLBCL. The number of patients that had relapsed disease was 15 (25%), while rituximab, cyclophosphamide, doxorubicin, vincristine, and prednisone (R-CHOP) therapy was given to 50 (83.3%) patients and R-mini-CHOP to 9 (15%) patients. Clinical and laboratory parameters for patients with DLBCL in our study is provided in Table 1.

**TABLE 1:** Baseline characteristics of the 60 patients included in the study.

Variable	Number of patients, n (%)
Sex	
Male	33 (55.0%)
Female	27 (45.0%)
Age (years)	
Median (range)	68.5 (24–86)
Age ≤60	21 (35.0%)
Age >60	21 (35.0%)
Stage	
Stage I and II	29 (48.3%)
Stage III and IV	30 (50.0%)
Missing	1 (1.7%)
R-IPI	
Low (0, 1)	23 (38.3%)
Intermediate (2)	15 (25.0%)
High (3, 4)	21 (35.0%)
Missing	1 (1.7%)
LDH (U/L)	
LDH ≤250	26 (43.3%)
LDH >250	33 (55.0%)
Missing	1 (1.7%)
NLR	
NLR ≤5.2	46 (76.7%)
NLR >5.2	13 (21.7%)
Missing	1 (1.7%)
LMR	
LMR ≤2.1	20 (33.3%)
LMR >2.1	39 (65.0%)
Missing	1 (1.7%)
Outcome	
Dead	18 (30%)
Censored (alive)	42 (70%)
COO	
ABC-like	21 (35.6%)
GCB-like	13 (22.0%)
NOS	25 (42.4%)

Abbreviations: ABC-like: activated B-cell-like; COO: cell-of-origin; GCB-like: germinal center B-cell-like; LDH: lactate dehydrogenase; LMR: lymphocyte-to-monocyte ratio; NLR: neutrophil-to-lymphocyte ratio; R-IPI: revised international prognostic index.

In total, we found 75 different low-resolution HLA molecules in patients with DLBCL and controls. More specifically, we detected 16 different HLA-A, 24 different HLA-B, 14 different HLA-C, seven different HLA-DQB1, and 13 different HLA-DRB1 molecules in patients with DLBCL and control subjects (Supplementary Table S1).

### 3.2 Associations between HLA class I and DLBCL risk

Thirteen different HLA-A molecules were detected in patients with DLBCL, *versus* sixteen in the control group, with no statistically significant difference between the groups. The additional three different HLA-A molecules that were detected in the control group only, were HLA-A*25, HLA-A*31, and HLA-A*66. More specifically, HLA-A*25 was detected in 7 (1.49%), HLA-A*31 in 10 (2.13%), and HLA-A*66 in 4 (0.85%) healthy controls (Table 2). The total number of HLA-B alleles detected in the control and DLBCL groups were 23 and 21, respectively, with no statistically significant difference in the phenotypic frequency of the HLA molecules between the groups, with the exception of HLA-B*39, the frequency of which was significantly lower in patients with DLBCL compared with controls (Table 2). The total number of HLA-C alleles detected in the control and DLBCL groups was 13 and 14, respectively. The phenotypic frequency of HLA-C*12 was significantly lower in the DLBCL group compared to the control population (3.33% vs. 17.9%, OR = 0.16, 95% CI: 0.04–0.44) (Table 2).

**TABLE 2:** Distribution of HLA-A, -B, and -C alleles in patients with DLBCL and controls.

Allele	Controls (2N = 472)	Controls, % (2N = 472)	Patients (2N = 120)	Patients, % (2N = 120)	*p*-value	OR	95% CI
HLA-A							
A*01	43	9.15	5	4.17	0.09	0.43	[0.13–1.12]
A*02	148	31.49	40	33.33	0.74	1.09	[0.69–1.7]
A*03	50	10.64	12	10	1	0.93	[0.44–1.86]
A*11	34	7.23	9	7.5	0.85	1.04	[0.43–2.3]
A*23	12	2.55	3	2.5	1	0.98	[0.17–3.71]
A*24	60	12.77	19	15.83	0.37	1.28	[0.69–2.3]
A*25	7	1.49	0	0	0.35	0.26	[0.01–4.52]
A*26	32	6.81	4	3.33	0.2	0.47	[0.12–1.37]
A*29	10	2.13	3	2.5	0.73	1.18	[0.21–4.68]
A*30	6	1.28	4	3.33	0.13	2.66	[0.54–11.43]
A*31	10	2.13	0	0	0.23	0.18	[0.01–3.13]
A*32	28	5.96	7	5.83	1	0.98	[0.35–2.37]
A*33	7	1.49	5	4.17	0.08	2.87	[0.7–10.72]
A*66	4	0.85	0	0	0.59	0.43	[0.02–8.04]
A*68	18	3.83	8	6.67	0.21	1.8	[0.66–4.47]
A*69	1	0.21	1	0.83	0.37	3.93	[0.05–309.22]
HLA-B							
B*07	16	3.43	3	2.5	0.78	0.72	[0.13–2.58]
B*08	14	3	4	3.33	0.77	1.11	[0.26–3.63]
B*13	15	3.22	5	4.17	0.58	1.31	[0.36–3.88]
B*14	9	1.93	5	4.17	0.18	2.2	[0.57–7.49]
B*15	13	2.79	4	3.33	0.76	1.2	[0.28–3.98]
B*18	53	11.37	14	11.67	0.87	1.03	[0.51–1.97]
B*27	13	2.79	0	0	0.08	0.14	[0.01–2.36]
B*35	80	17.17	21	17.5	0.89	1.02	[0.57–1.77]
B*37	15	3.22	1	0.83	0.21	0.25	[0.01–1.68]
B*38	17	3.65	2	1.67	0.39	0.45	[0.05–1.93]
B*39	17	3.65	0	0	0.03	0.11	[0.01–1.79]
B*40	13	2.79	6	5	0.25	1.83	[0.56–5.3]
B*41	7	1.5	1	0.83	1	0.55	[0.01–4.36]
B*44	31	6.65	13	10.83	0.12	1.7	[0.79–3.49]
B*47	1	0.21	2	1.67	0.11	7.84	[0.41–464.48]
B*48	0	0	1	0.83	0.2	11.7	[0.47–289.3]
B*49	11	2.36	5	4.17	0.34	1.8	[0.48–5.74]
B*50	8	1.72	2	1.67	1	0.97	[0.1–4.95]
B*51	86	18.45	21	17.5	0.89	0.94	[0.53–1.62]
B*52	13	2.79	0	0	0.08	0.14	[0.01–2.36]
B*55	15	3.22	3	2.5	1	0.77	[0.14–2.79]
B*56	3	0.64	1	0.83	1	1.3	[0.02–16.31]
B*57	11	2.36	3	2.5	1	1.06	[0.19–4.10]
B*58	5	1.07	3	2.5	0.12	2.36	[0.36–12.33]
HLA-C							
C*01	19	4.15	6	5	0.62	1.22	[0.39–3.26]
C*02	29	6.33	10	8.33	0.42	1.34	[0.57–2.94]
C*03	23	5.02	11	9.17	0.12	1.91	[0.81–4.22]
C*04	78	17.03	24	20	0.5	1.22	[0.7–2.07]
C*05	10	2.18	6	5	0.11	2.35	[0.69–7.32]
C*06	44	9.61	11	9.17	1	0.95	[0.43–1.95]
C*07	82	17.9	26	21.67	0.36	1.27	[0.74–2.12]
C*08	10	2.18	6	5	0.11	2.35	[0.69–7.32]
C*12	82	17.9	4	3.33	1.11E-05	0.16	[0.04–0.44]
C*14	17	3.71	4	3.33	1	0.89	[0.21–2.81]
C*15	37	8.08	8	6.67	0.7	0.81	[0.32–1.84]
C*16	22	4.8	2	1.67	0.2	0.34	[0.04–1.4]
C*17	5	1.09	1	0.83	1	0.76	[0.02–6.9]
C*18	0	0	1	0.83	0.2	11.51	[0.47–284.35]

*p* values < 0.05 were considered statistically significant.

Abbreviations: CI: confidence interval; DLBCL: diffuse large B-cell lymphoma; N: number; OR: odds ratio.

### 3.3 Associations between HLA class II and DLBCL risk

Moreover, HLA-II allele frequencies were calculated and compared between the two groups. Thirteen different low-resolution HLA-DRB1 molecules were detected in patients with DLBCL and controls, with no statistically significant difference in the phenotypic frequency of the HLA molecules between the groups (Table 3). Finally, seven different low-resolution HLA-DQB1 molecules were detected in both control and DLBCL groups with no statistically significant difference in the phenotypic frequency of the HLA molecules between the groups (Table 3).

**TABLE 3:** Distribution of HLA-DQB1 and -DRB1 alleles in patients with DLBCL and controls.

Allele	Control (2N = 472)	Controls, % (2N = 472)	Patient (2N = 120)	Patients, % (2N = 120)	*p*-value	OR	95% CI
HLA-DQB1							
DQB1*02	46	10.9	13	11.82	0.49	1.24	[0.59–2.45]
DQB1*04	8	1.9	2	1.82	1	0.96	[0.1–4.9]
DQB1*05	123	29.15	32	29.09	1	1	[0.61–1.61]
DQB1*06	62	14.69	16	14.55	1	0.99	[0.51–1.83]
DQB1*03:01 (DQ7)	141	33.41	35	31.82	0.8	0.93	[0.57–1.49]
DQB1*03:02 (DQ8)	29	6.87	10	9.09	0.4	1.35	[0.57–2.98]
DQB1*03:03 (DQ9)	6	1.42	2	1.82	0.67	1.28	[0.13–7.31]
HLA-DRB1							
DRB1*01	27	5.97	13	10.83	0.07	1.91	[0.87–3.98]
DRB1*03	24	5.31	7	5.83	0.82	1.1	[0.39–2.73]
DRB1*04	43	9.51	13	10.83	0.72	1.16	[0.55–2.29]
DRB1*07	27	5.97	10	8.33	0.4	1.43	[0.6–3.16]
DRB1*08	12	2.65	3	2.5	1	0.94	[0.17–3.56]
DRB1*09	2	0.44	1	0.83	0.51	1.89	[0.03–36.57]
DRB1*10	11	2.43	1	0.83	0.48	0.34	[0.01–2.36]
DRB1*11	123	27.21	34	28.33	0.82	1.06	[0.65–1.68]
DRB1*12	11	2.43	2	1.67	1	0.68	[0.07–3.18]
DRB1*13	45	9.96	12	10	1	1	[0.47–2.02]
DRB1*14	26	5.75	9	7.5	0.52	1.33	[0.53–3.03]
DRB1*15	34	7.52	5	4.17	0.23	0.53	[0.16–1.42]
DRB1*16	67	14.82	10	8.33	0.07	0.52	[0.23–1.07]

*p* values < 0.05 were considered statistically significant.

Abbreviations: CI: confidence interval; DLBCL: diffuse large B-cell lymphoma; N: number; OR: odds ratio.

### 3.4 Notable differences in HLA allele frequency between DLBCL patients and controls that did not reach statistical significance

The alleles that slightly differed between DLBCL patients and controls were HLA-Α*01 (9.15% [controls] vs. 4.17% [patients], *p* = 0.09, OR = 0.43, 95% CI [0.13–1.12]), HLA-Α*33 (1.49% [controls] vs. 4.17% [patients], *p* = 0.08, OR = 2.87, 95% CI [0.7–10.72]), HLA-Β*27 (2.79% [controls] vs. 0% [patients], *p* = 0.08, OR = 0.14, 95% CI [0.01–2.36]), HLA-Β*52 (2.79% [controls] vs. 0% [patients], *p* = 0.08, OR = 0.14, 95% CI [0.01–2.36]), HLA-DRB1*01 (5.97% [controls] vs. 10.83% [patients], *p* = 0.07, OR = 1.91, 95% CI [0.87–3.98]), and HLA-DRB1*16 (14.82% [controls] vs. 8.33% [patients], *p* = 0.07, OR = 0.52, 95% CI [0.23–1.07]) (Supplementary Table S2).

### 3.5 Survival analysis results

Univariate survival analysis indicated statistically significant impact of stage, R-IPI and LDH both in the OS (*p*-value = 0.007, *p*-value = 0.002 and *p*-value = 0.003, respectively) (Supplementary Figure S1) and the PFS (*p*-value = 0.001, *p*-value = 2.45E-04 and *p*-value = 0.002, respectively) (Supplementary Figure S2). Statistically significant results were not found for the other variables (Supplementary Figures S1 and S2). Results of non-significant factors, both for OS and PFS, are depicted in Supplementary Table S3.

**FIGURE 1:**
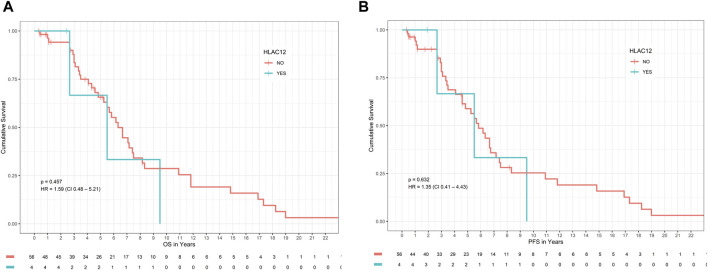
Impact of HLA-C*12 status on **(A)** OS and **(B)** PFS in patients with DLBCL. Abbreviations: DLBCL: Diffuse large B-cell lymphoma; HLA: Human leucocyte antigen; OS: overall survival; PFS: progression-free survival.

Presence of allele HLA-C*12 which was found to be significantly associated with DLBCL risk in our study, was not associated with significantly different OS or PFS compared with patients with absence of HLA-C*12, *p*-value >0.05 (Figure 1). Specifically, the median OS of patients with the presence of allele HLA-C*12 (4 patients with percentage 6.7%) was 5.5 years (HR = 1.42 with 95% CI [0.43–4.63]), whereas in patients with absence of HLA-C*12 was 6.2 years (0.71 with 95% CI [0.22–2.31]), *p*-value = 0.57 > 0.05. The median PFS for patients with the presence of HLA-C*12 was 5.7 years (HR = 1.22 with 95% CI [ [0.37–3.97]), whereas for patients with absence of HLA-C*12 was 5.5 years (HR = 0.82 with 95% CI [0.25–2.68]), *p*-value = 0.76 > 0.05. In multivariate Cox proportional hazards analysis (where we included the HLA-C*12 allele and statistically significant variables found in univariate analysis), the *p*-values were both statistically significant for OS (*p*-value = 0.008 < 0.05) and PFS (*p*-value = 0.001 < 0.05), indicating that the models are significant. However, co-variables stage, R-IPI LDH and the HLA-C*12, failed to be significant prognostic factors affecting survival in both models (*p*-values >0.05), as described in Table 4.

**TABLE 4:** Multivariate analysis models for OS and PFS in DLBCL patients, including stage, R-IPI, LDH, and HLA-C*12 status.

Multivariate analysis	OS			PFS		
Variables	Hazard ratio	95%CI	*p*-value	Hazard ratio	95%CI	*p*-value
Stage (Stage III and IV)	1.12	(0.37–3.39)	0.84	1.04	(0.35–3.08)	0.95
R-IPI: Intermediate	1.52	(0.58–4.01)	0.4	0.91	(0.34–2.38)	0.84
R-IPI: Low	0.44	(0.10–1.88)	0.27	0.22	(0.05–0.97)	0.05
LDH: Normal (LDH ≤250 I/U)	0.47	(0.22–1.04)	0.06	0.54	(0.24–1.19)	0.13
HLA-C*12: YES	0.81	(0.22–3.01)	0.76	0.46	(0.12–1.81)	0.26

*p* values < 0.05 were considered statistically significant.

Abbreviations: CI: confidence interval; DLBCL: Diffuse large B-cell lymphoma risk; HLA: human leucocyte antigen; LDH: lactate dehydrogenase; OS: Overall survival: PFS: Progression-free survival; R-IPI: revised international prognostic index.

## 4 Discussion

In the present study, we analyzed the phenotypic frequencies of HLA-A, -B, -C, -DRB1, and -DQB1 allele polymorphisms in 60 DLBCL patients and 236 healthy control individuals. From our results, HLA-C*12 was significantly lower in patients with DLBCL compared with control subjects. Next, in an effort to ascertain the impact of HLA variations on DLBCL prognosis, we used univariate and multivariate analyses to assess whether the presence of HLA-C*12, in conjunction with other parameters that have been found to affect DLBCL prognosis, could affect OS and PFS. Results revealed that HLA-C*12 was not significantly associated with varying DLBCL prognosis, both in univariate analysis and when used in multivariate models.

Although our study did not find significant associations between HLA-A and -B alleles and risk of DLBCL, other studies have found significant associations regarding these molecules. More specifically, Alcoceba et al. (Alcoceba et al., 2013) compared 250 DLBCL cases to 1940 controls of European origin and found that the phenotypic frequency of HLA-DRB1*01 was higher (29% vs. 19.5%, OR = 1.69, *p*-value = 0.0008) in DLBCL patients than in the control group. Our study found similar results for DRB1*01 in patients and controls but without statistical significance. The phenotypic frequency of HLA-B*51 was also higher in the DLBCL group in a study conducted by Choi et al. (Choi et al., 2008), who assessed a Korean population of 89 DLBCL cases and 200 controls. The same study also concluded that HLA-A*33 and HLA-B*44 occurred less frequently in DLBCL patients compared to controls. Moreover, Cernhan et al. (Cerhan et al., 2014) conducted a meta-analysis of three GWAS, including 3857 DLBCL cases and 7666 controls of European ancestry. In this study, genome-wide significance was achieved for HLA-B*08:01 (*p*-value = 3.16 × 10^−8^), which was found to be a major determinant of DLBCL risk. Finally, Wang *et al.* (Wang et al., 2010) compared 610 NHL cases and 555 controls of non-Hispanic white descent in the US, with HLA-A*26:01 and HLA-B*51:01 increasing DLBCL risk.

It needs to be emphasized that, although HLA-B*39 was found to be significantly lower in patients with DLBCL compared with controls, this polymorphism is very rare among Greek individuals (Arnaiz-Villena et al., 2001), and, given the small sample size of our study, conclusions regarding its significance are limited.

According to our results, the frequency of HLA-C*12 was lower in DLBCL patients compared to the control group. HLA-C alleles have been implicated in the pathogenesis of many diseases, such as rheumatoid arthritis, Crohn’s disease (Siegel et al., 2019), as well as malignancies, such as leukemia and lymphoma (Mohammad et al.). The HLA-C alleles serve as ligands for the killer-cell immunoglobulin-like receptor (KIR) family, which is a highly polymorphic gene region, the products of which are heavily expressed by natural killer (NK) cells. Variations of HLA-C and/or KIR alleles have been associated with various disease processes and responses to viral infections, with some studies also reporting an association of specific HLA-C/KIR gene variations with cancer risk. Since NK cells are implicated in the antitumoral immune response, this could indicate that HLA-C gene variations, with or without accompanying KIR polymorphisms, could affect cancer risk development (Vollmers et al., 2021; Hematian et al., 2022). Regarding DLBCL, Alcoceba et al. (Alcoceba et al., 2013) and Wang *et al.* (Wang et al., 2010) have found that the phenotypic frequency of HLA-C*03 is lower and HLA-Cw*15:02 is higher in the DLBCL group compared to controls, respectively. More studies will need to be conducted to truly delineate the specific role of HLA-C on DLBCL risk, especially by taking into consideration the interaction between this molecule and components of the innate immune system.

Our results revealed that, on univariate analysis, HLA-C*12 was not significantly associated with OS and PFS in DLBCL patients. However, published studies have found specific HLA variations that impact survival in patients with DLBCL. Specifically, Alcoceba et al. (Alcoceba et al., 2013) studied 250 DLBCL cases and 1940 controls, and revealed that patients receiving R-CHOP regimen and carrying the HLA-B44 supertype (and especially the HLA-B*18 polymorphism) had a worse 5-year PFS (54% vs. 71%, *p*-value = 0.019) and OS (71% vs. 92%, *p*-value = 0.001). Next, Lu et al. (Lu et al., 2011) assessed 166 DLBCL patients and found that the presence of HLA-Cw*07:01 allele was associated with worse OS (HR = 1.76, 95% CI [1.24, 4.01]). Since survival analysis was a secondary outcome in our study, we studied only HLA variations that were significantly associated with DLBCL development for prognostic outcomes, and, as such, HLA alleles that were not accounted for in our survival analysis might be associated with varying OS/PFS in patients with DLBCL. Finally, even though, in multivariate analysis, our models did find statistically significant results, it is important for future studies to incorporate HLA variations in their analyses together with other parameters, such as stage, R-IPI, and biological variables, that have been shown to affect prognosis in patients with DLBCL.

Our study has certain limitations. The inclusion of only Caucasian individuals limits the generalizability of our study results. The age range of our patients was wide, specifically 18–85 years (median age 68.5). Also, we did not include individuals with autoimmune diseases in our patient cohort, since this group of patients is at increased risk for the development of malignancies, and thus could act as a confounding variable that could distort the relationship between HLA variations and DLBCL risk. Genetic subgroup analysis was not available in our patients, and, as such, further stratification according to genetic subgroups did not occur. HLA allele distribution in patients with GCB DLBCL *versus* non-GCB DLBCL was also not available in our analysis. It also needs to be mentioned that, due to small sample size, survival analysis could only be undertaken for HLA alleles found to be significantly associated with DLBCL development, and not for all HLA polymorphisms studied. Next, except for healthy donor median age, sex, and underlying comorbidities that would exclude them from potential bone marrow donors, we were unable to extract further data to characterize the control group, and, as such, multivariable analyses to account for potential inter-group confounders was not available. Finally, the sample size of the control group was relatively small, compared to other studies conducted to assess HLA polymorphisms in patients with DLBCL, which may have prevented us from finding significant associations with DLBLC development that have been previously described in the literature.

## 5 Conclusion

HLA variations have been well described in the literature for affecting DLBCL development. This observation reinforces the genetic component of the disease, although more research is still required to delineate the specific gene associations linked to DLBCL development. Whole-genome studies have revealed new HLA alleles directly related to DLBCL, although the interpretation of these findings needs to be made cautiously. Restrictions regarding the study population are evident in some studies, and generalization of the results to populations of different ethnicity may lead to erroneous associations that are not substantiated. Although some HLA polymorphisms are not ethnicity-specific, others are closely related to the origin of the study population, which may also account for the varying incidence rate of DLBCL according to the reference population. Also, linkage disequilibrium needs to be taken into consideration, since the impact of an HLA variation on DLBCL risk may be attributed to another HLA polymorphism, or to non-HLA genes. Nevertheless, the growing number of HLA alleles that contribute to lymphoma risk may aid in a better understanding of disease pathogenesis, with implications in both the treatment process, as well as prognosis. Prospective, well-designed studies are required to ascertain the impact of HLA variations on DLBCL development and prognosis while taking into account already established parameters that affect OS/PFS in these patients.

## Data Availability

The datasets presented in this study can be found in online repositories. The names of the repository/repositories and accession number(s) can be found in the article/Supplementary Material.
