# Supplementary material for: HLA variations in patients with diffuse large B-cell lymphoma and association with disease risk and prognosis: a case-control study
Source: Front Genet. 2024 Apr 12;15:1341822. doi: 10.3389/fgene.2024.1341822 (PMC11045888; doi:10.3389/fgene.2024.1341822)
Supplement: Supplementary file 1 [file DataSheet1.docx]

**Supplementary Appendix**

Supplement to: HLA Variations and Association with Diffuse Large B-Cell Lymphoma Risk

**Contents**

Section 1: Supplemental Tables and Figures

**Table S1:** HLA-A, -B, -C, -DRB1 and -DQB1 phenotypic frequencies in patients with DLBCL compared to patients in the control group.

**Table S2:** Notable differences in HLA allele frequency between DLBCL patients and controls that did not reach statistical significance.

**Table S3:** Univariate analysis of variables included in the survival analysis.

**Figure S1:**  Univariate analysis **(A)** age, **(B)** COO, **(C)** LDH, **(D)** LMR, **(E)** NLR, **(F)** R-IPI, **(G)** Relapse status, **(H)** sex, **(I)** stage and **(J)** treatment in the overall survival of patients with DLBCL. Abbreviations: ABC: Activated B-cell like; COO: cell-of-origin; DLBCL: Diffuse large B-cell lymphoma; GCB: Germinal center B-cell like; LMR: Lymphocyte-to-monocyte ratio; NLR: neutrophil-to-lymphocyte ratio; NOS: not otherwise specified; R-IPI: Revised International Prognostication Index.

**Figure S2:**  Univariate analysis **(A)** age, **(B)** COO, **(C)** LDH, **(D)** LMR, **(E)** NLR, **(F)** R-IPI, **(G)** Relapse status, **(H)** sex, **(I)** stage and **(J)** treatment in the progression-free survival of patients with DLBCL. Abbreviations: ABC: Activated B-cell like; COO: cell-of-origin; DLBCL: Diffuse large B-cell lymphoma; GCB: Germinal center B-cell like; LMR: Lymphocyte-to-monocyte ratio; NLR: neutrophil-to-lymphocyte ratio; NOS: not otherwise specified; R-IPI: Revised International Prognostication Index.

| **Table S1:** HLA-A, -B, -C, -DRB1 and -DQB1 phenotypic frequencies in patients with DLBCL compared to patients in the control group. | | | | | | | | | | | |
| --- | --- | --- | --- | --- | --- | --- | --- | --- | --- | --- | --- |
| **HLA phenotype** | **Control 2n = 472 %** | **Patients 2n = 120 %** | **P** | **HLA phenotype** | **Control 2n = 472 %** | **Patients 2n = 120 %** | **P** | **HLA phenotype** | **Control 2n = 472 %** | **Patients 2n = 120 %** | **P** |
|  |  |  |  |  |  |  |  |  |  |  |  |
| A*01 | 9.15 | 4.17% | NS | B*38 | 3.65% | 1.67% | NS | C*15 | 8.08% | 6.67% | NS |
| A*02 | 31.49 | 33.33% | NS | B*39 | 3.65% | 0% | <.001 | C*16 | 4.80% | 1.67% | NS |
| A*03 | 10.64 | 10% | NS | B*40 | 2.79% | 5% | NS | C*17 | 1.09% | 0.83% | NS |
| A*11 | 7.23 | 7.50% | NS | B*41 | 1.50% | 0.83% | NS | C*18 | 0% | 0.83% | NS |
| A*23 | 2.55 | 2.50% | NS | B*44 | 6.65% | 10.83% | NS |  |  |  |  |
| A*24 | 12.77% | 15.83% | NS | B*47 | 0.21% | 1.67% | NS | DQB1*02 | 10.90% | 11.82% | NS |
| A*25 | 1.49% | 0% | NS | B*48 | 0% | 0.83% | NS | DQB1*04 | 1.90% | 1.82% | NS |
| A*26 | 6.81% | 3.33% | NS | B*49 | 2.36% | 4.17% | NS | DQB1*05 | 29.15% | 29.09% | NS |
| A*29 | 2.13% | 2.50% | NS | B*50 | 1.72% | 1.67% | NS | DQB1*06 | 14.69% | 14.55% | NS |
| A*30 | 1.28% | 3.33% | NS | B*51 | 18.45% | 17.50% | NS | DQB1*03:01 (DQ7) | 33.41% | 31.82% | NS |
| A*31 | 2.13% | 0% | NS | B*52 | 2.79% | 0% | NS | DQB1*03:02 (DQ8) | 6.87% | 9.09% | NS |
| A*32 | 5.96% | 5.83% | NS | B*55 | 3.22% | 2.50% | NS | DQB1*03:03 | 1.42% | 1.82% | NS |
|  |  |  |  |  |  |  |  | (DQ9) |  |  |  |
| A*33 | 1.49% | 4.17% | NS | B*56 | 0.64% | 0.83% | NS |  |  |  |  |
| A*66 | 0.85% | 0% | NS | B*57 | 2.36% | 2.50% | NS | DRB1*01 | 5.97% | 10.83% | NS |
| A*68 | 3.83% | 6.67% | NS | B*58 | 1.07% | 2.50% | NS | DRB1*03 | 5.31% | 5.83% | NS |
| A*69 | 0.21% | 0.83% | NS |  |  |  |  | DRB1*04 | 9.51% | 10.83% | NS |
|  |  |  |  | C*01 | 4.15% | 5% | NS | DRB1*07 | 5.97% | 8.33% | NS |
| B*07 | 3.43% | 2.50% | NS | C*02 | 6.33% | 8.33% | NS | DRB1*08 | 2.65% | 2.50% | NS |
| B*08 | 3% | 3.33% | NS | C*03 | 5.02% | 9.17% | NS | DRB1*09 | 0.44% | 0.83% | NS |
| B*13 | 3.22% | 4.17% | NS | C*04 | 17.03% | 20% | NS | DRB1*10 | 2.43% | 0.83% | NS |
| B*14 | 1.93% | 4.17% | NS | C*05 | 2.18% | 5% | NS | DRB1*11 | 27.21% | 28.33% | NS |
| B*15 | 2.79% | 3.33% | NS | C*06 | 9.61% | 9.17% | NS | DRB1*12 | 2.43% | 1.67% | NS |
| B*18 | 11.37% | 11.67% | NS | C*07 | 17.90% | 21.67% | NS | DRB1*13 | 9.96% | 10% | NS |
| B*27 | 2.79% | 0% | NS | C*08 | 2.18% | 5% | NS | DRB1*14 | 5.75% | 7.50% | NS |
| B*35 | 17.17% | 17.50% | NS | C*12 | 17.90% | 3.33% | <.001 | DRB1*15 | 7.52% | 4.17% | NS |
| B*37 | 3.22% | 0.83% | NS | C*14 | 3.71% | 3.33% | NS | DRB1*16 | 14.82% | 8.33% | NS |
| Abbreviations: DLBCL: diffuse large B-cell lymphoma; HLA: human leukocyte antigen; NS: not significant. | | | | | | | | | | | |

| **Table S2:** Notable differences in HLA allele frequency between DLBCL patients and controls that did not reach statistical significance | | | | | | | |
| --- | --- | --- | --- | --- | --- | --- | --- |
| **Allele** | **Control (2N=472)** | **Controls, % (2N=472)** | **Patient (2N=120)** | **Patients, % (2N=120)** | **P-value** | **OR** | **95%CI** |
| HLA-A*01 | 43 | 9.15 | 5 | 4.17 | 0.09 | 0.43 | [0.13-1.12] |
| HLA-A*33 | 7 | 1.49 | 5 | 4.17 | 0.08 | 2.87 | [0.7-10.72] |
| HLA-B*27 | 13 | 2.79 | 0 | 0 | 0.08 | 0.14 | [0.01-2.36] |
| HLA-B*52 | 13 | 2.79 | 0 | 0 | 0.08 | 0.14 | [0.01-2.36] |
| HLA-DRB1*01 | 27 | 5.97 | 13 | 10.83 | 0.07 | 1.91 | [0.87-3.98] |
| HLA-DRB1*16 | 67 | 14.82 | 10 | 8.33 | 0.07 | 0.52 | [0.23-1.07] |
| Abbreviations: CI: confidence interval; DLBCL: diffuse large B-cell lymphoma; N: number; OR: odds ratio. | | | | | | | |

**Table S3:** Univariate analysis of variables included in the survival analysis.




**Figure S1**

**
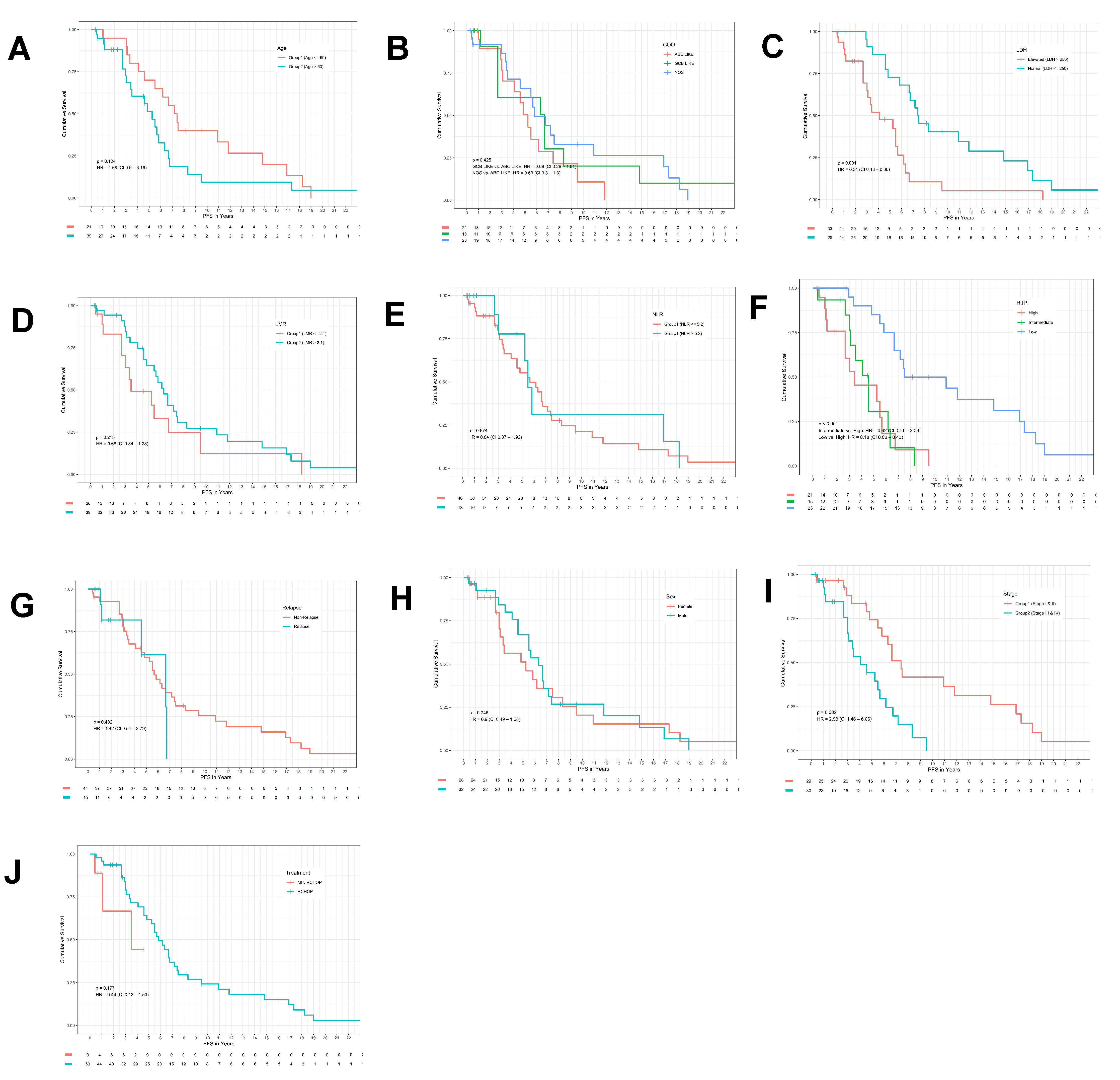
**

**Figure S2**
